# A novel panel of monoclonal antibodies against Schmallenberg virus nucleoprotein and glycoprotein Gc allows specific orthobunyavirus detection and reveals antigenic differences

**DOI:** 10.1186/s13567-015-0165-4

**Published:** 2015-03-11

**Authors:** Kerstin Wernike, Emiliana Brocchi, Paolo Cordioli, Yann Sénéchal, Christian Schelp, Anne Wegelt, Andrea Aebischer, Gleyder Roman-Sosa, Ilona Reimann, Martin Beer

**Affiliations:** Institute of Diagnostic Virology, Friedrich-Loeffler-Institut (FLI), Suedufer 10, 17493 Greifswald - Insel Riems, Germany; Istituto Zooprofilattico Sperimentale della Lombardia e dell’Emilia Romagna, Via Bianchi 7, 25125 Brescia, Italy; IDEXX Switzerland AG, Stationsstrasse 12, 3097 Liebefeld-Bern, Switzerland

## Abstract

A panel of monoclonal antibodies (mAbs) specific for the nucleocapsid (N) protein or the glycoprotein Gc of Schmallenberg virus (SBV), a novel member of the Simbu serogroup (genus *Orthobunyavirus*, family *Bunyaviridae*), was produced and used to analyze antigenic differences among members of this serogroup. Reactivity with various SBV-isolates and other Simbu serogroup viruses was assessed by an indirect immunofluorescence test and by immunoblotting. The Gc-specific mAbs detected different SBV isolates as well as two closely related members of the Simbu serogroup. In addition, one mAb showed a highly specific reactivity with the homologous SBV strain only. Based on their differing reactivity with different SBV-strains, these antibodies represent a valuable novel tool to rapidly determine the phenotype of new SBV isolates. In contrast, the N-specific mAbs showed a broad reactivity spectrum and detected not only all the tested SBV-isolates, but also several other viruses of the Simbu serogroup. One out of these mAbs even recognized all of the tested Simbu serogroup viruses in the indirect immunofluorescence assay. In order to further characterize the N-specific antibodies, PepScan analysis was performed and a specific epitope could be identified. In summary, the newly generated mAbs showed differing pan-Simbu virus-, pan-SBV- as well as SBV-isolate-specific reactivity patterns. Thus, they represent valuable tools for the development of novel antigen and antibody detection systems either specific for SBV or, in a broader approach, for the pan-Simbu serogroup diagnostics.

**Electronic supplementary material:**

The online version of this article (doi:10.1186/s13567-015-0165-4) contains supplementary material, which is available to authorized users.

## Introduction

In late 2011, a hitherto unknown teratogenic orthobunyavirus was discovered in Central Europe and named Schmallenberg virus (SBV) [1]. It causes severe congenital malformation, stillbirth or premature birth, when pregnant ruminants are infected during a critical period of gestation. SBV is a member of the genus *Orthobunyavirus* within the family *Bunyaviridae* [1]. Orthobunyaviruses are divided into 18 serogoups and among these SBV belongs to the Simbu serogroup [2,3]. The closest relatives of SBV are Sathuperi virus (SATV) and Douglas virus (DOUV) [2]. However, the Simbu serogroup also includes Akabane virus (AKAV) and Aino virus (AINOV), as well as Oropouche virus (OROV), which is the only member of the serogroup capable to infect humans.

Like in typical bunyaviruses, the SBV genome consists of three single-stranded RNA segments which encode six proteins. According to their size they are named large (L), medium (M) and small (S). The nucleocapsid (N) protein and a small non-structural protein (NSs) are encoded in overlapping open reading frames by the S segment, two glycoproteins (Gn and Gc), as well as a non-structural protein (NSm) are encoded by the M segment, and the RNA-dependent RNA polymerase which represents the central element of the orthobunyaviral replication complex [4] is encoded by the L segment [5,6].

The N‐protein has a molecular weight of 25 kDa, oligomerizes as a tetramer [7], and is not only essential for viral genome encapsidation, but is also involved in viral RNA‐transcription and replication [7,8]. Furthermore, it is the most conserved protein among orthobunyaviruses, and elicits a strong humoral immune response in infected animals [9-11]. The glycoproteins Gn and Gc with molecular masses of approximately 35 kDa and 110 kDa, respectively, represent type I integral transmembrane proteins which are further modified by N-linked glycosylation [8]. They form spikes on the virus particle and are essential for viral attachment and cell fusion. It has been described before for certain bunyaviruses, that Gn and Gc are targeted by neutralizing antibodies [5]. Similar roles are also assumed for the SBV Gn and Gc, but remain to be confirmed. In contrast to the nucleocapsid protein, the glycoproteins, especially Gc, show the most variable sequences among the protein-coding genes of SBV and related viruses [12-15].

Currently, molecular and serological detection systems for SBV primarily base on the N-protein [16,17]. In the present study, monoclonal antibodies (mAb) specific for N as well as for Gc were prepared and characterized. Special emphasis was set on the investigation of the suitability of the mAbs for application in SBV diagnostics and for the characterization of virus isolates.

## Materials and methods

### Virus purification

SBV, strain BH80/11, was propagated in Vero cell monolayers and harvested when the cytopathic effect (CPE) was maximal; supernatant fluid was clarified by centrifugation at 4000 rpm for 30 min, added to 8% of PEG 6000 in 0.5 M NaCl, and then placed overnight at 4 °C in agitation. The suspension was centrifuged at 5000 rpm for 30 min and the pellet was resuspended in phosphate-buffer saline solution, pH 7.4 (PBS) at a 20X concentration compared to the initial volume. Following further clarification by centrifugation (5000 rpm for 30 min), the viral suspension was purified by ultracentrifugation at 35 000 rpm for 2 h (rotor TST41 Kontron) through a 25% (w/w) sucrose cushion and the pellet was resuspended in PBS. The concentrated antigen was kept at −70 °C until use.

### MAbs production

Two Balb/c mice were primed with intraperitoneal injections of 4 × 10^6^ BHK-21 cells infected with SBV strain BH80/11 (dilutions in PBS). After one month, mice were boosted as follows: one using again the initial antigen preparation, and the second one using partially purified virus (500 μg of total protein containing approximately 50 μg of viral proteins).

Three days after the boost, mice were humanely sacrificed and hybridomas were generated by fusion of splenocytes with NS0 myeloma cells following standard procedures [18]. Briefly, at least 10^8^ spleen cells were recovered from each mouse and fused with NS0 myeloma cells at a 10:1 ratio using PEG 4000. Fused cells diluted in Dulbecco’s modified Eagle medium, supplemented with hypoxanthine/aminopterin/thymidine and 20% fetal calf serum, were distributed over five microplates (200 μL per well). Growing colonies were observed in all wells; in order to select hybridomas secreting monoclonal antibodies specific for SBV, the supernatants were screened by indirect immunofluorescence (IIF) test using SBV-infected Vero cells, grown into 96-well microplates and fixed by 80% acetone. Non-infected Vero cells served as negative controls.

The positive hybridoma cells were cloned by limiting dilution in order to obtain antibodies from one single cell. The supernatant from exhausted cultures was then used as source of mAb.

### Indirect ELISA

ELISA was performed in 96-well Nunc Maxisorp ELISA plates. The plates were coated over night at 4 °C using 50 μL/well of partially purified SBV-antigen at a saturating concentration (produced as described above) diluted in ELISA coating buffer (0.05 M carbonate/bicarbonate buffer, pH 9.6). Plates were washed three times with 250 μL of washing buffer (PBS containing 0.05% Tween 20) and subsequently incubated with 50 μL/well of undiluted hybridoma culture supernatants for 1 h at 37 °C. After three washes, a peroxidase-conjugated goat anti-mouse immunoglobulin antibody (produced in-house) optimally diluted in PBS containing 0.05% Tween 20 and 1% yeast extract was incubated for 1 h at 37 °C. After a final washing cycle, 50 μL/well of substrate solution (0.5 mg/mL orthophenylenediamine and 0.02% H_2_O_2_ in 50 mM phosphate citrate buffer, pH 5) was added. The colorimetric reaction was stopped after 10 min using 2 N sulfuric acid and the absorbance values were read at 492 nm using an ELISA reader.

### Virus-neutralization test (VNT)

Serial two-fold dilutions of each hybridoma supernatant (duplicate wells) in 25 μL of serum-free culture medium were incubated for 1 h at 37 °C with an equal volume of cell culture supernatant containing 100 tissue culture infectious dose 50 (TCID_50_) of SBV. Subsequently, 50 μL of Vero cells at a dilution of 4 × 10^5^ cells/mL in medium containing 10% fetal calf serum were added to each well. After incubation for 72–96 h at 37 °C with 5% CO_2_, wells were scored for CPE and neutralizing titers were expressed as the reciprocal of the final mAb dilution required to neutralize 100% of the inoculated cultures.

### Immunofluorescence test

The hybridoma supernatants were characterized by an IIF-test using SBV-infected BHK-21 cells, clone BRS5 (L194, Collection of Cell Lines in veterinary Medicine [CCLV]) as antigen matrix [17]. The BHK-21 cells were infected with one of the following SBV strains: BH80/11 (isolated from bovine blood in 2011 on KC cells [L1062 CCLV]), BH619/12 (isolated from sheep serum in 2012 on Vero cells [L0228 CCLV]), BH652/12 (isolated from bovine blood in 2012 on BHK cells [L0164 CCLV]), D512/12, D495/12-1, and D495/12-2 (all three isolated in 2012 and kindly provided by Andreas Moss, Lebensmittel- und Veterinärinstitut Oldenburg, Germany). In addition, the mAbs were tested against closely related Simbu serogroup viruses, namely Simbu virus (SIMV), DOUV, SATV, Sabo virus (SABOV), Shamonda virus (SHAV), AKAV, AINOV, Peaton virus (PEAV), and OROV. All Simbu serogroup viruses were kindly provided by Peter Kirkland (Elizabeth Macarthur Agricultural Institute, Australia) and Robert Tesh (University of Texas Medical Branch, USA).

In addition, the mAbs were tested in DNA-transfected BSR-T7/5 cells (L583 CCLV, [19]), transiently expressing SBV-N, all M-segment encoded proteins (Gn, Gc, M) and Gn or Gc separately. For the construction of the expression plasmids pCITE_N, pCITE_M, pCITE_Gn and pCITE_Gc, the open reading frame (ORF) of SBV-N and SBV-M and the genomic regions encoding Gn and Gc, respectively, were amplified by RT-PCR from supernatants of SBV-infected BHK-21 cells. RNA was isolated by using the QIAmp viral RNA Mini Kit (Qiagen) and amplified with the QIAGEN OneStep RT-PCR Kit (Qiagen). Primers used for S were as follows: SBV_N_F (5’CCAATCTAGACCGATGTTGATACCGAATTGCTG 3’), SBV_N_R (3’ GAGTGCGGCCGCTTTAGATGTTGATACCGAATTG 5’), for M: SBV_Gn_F (5’ GAAAAACACGATGATAATACCATGCTTCTCAACATTGTCTTG 3’), SBV_Gc_R (3’ GTGGTGCTCGAGTGCGGCCGCTCACTT AAATTT ATTTTCCATTT GG 5’), for Gn: SBV_Gn_F and SBV_Gn_R (3’ GTGGTGCTCGAGTGCGGCCGCTT ACCTTGTTTTCG GCAATGCT TTATAG 5’) and for Gc: SBV_Gc_F (5’ GAAAAACA CGATGATAATACCATGGAAACTAG TATT AACTGCAAAAACA 3’) and SBV_Gc_R. The RT-PCR fragments were cloned into plasmid pCITE-2a (Novagen). After XbaI/NcoI digestion, the PCR fragment of SBV-N was ligated into the NcoI/XbaI digested plasmid resulting in plasmid pCITE_N. Plasmid constructs pCITE_M, pCITE_Gn and pCITE_Gc were generated by restriction-free cloning [20]. For protein expression, plasmid-DNA was transfected into BSR-T7/5 cells which stably express the phage T7 RNA polymerase [19] using Lipofectamine 2000 (Invitrogen) according to manufacturer’s protocols. At 24 h post transfection, the transfected cells were used for the investigation of the mAbs by IIF-staining.

### SDS–PAGE and Western blot analysis

The reactivity of the hybridoma supernatants with the SBV-strains mentioned above as well as with SIMV, DOUV, SATV, SABOV, SHAV, AKAV, AINOV, and OROV was additionally analyzed by Western blot (WB). The viruses were propagated on BHK-cells (L0164 CCLV) and were deep frozen at −70 °C when a generalized cytopathic effect became evident after 24 – 48 h post infection. Uninfected BHK-cells were used as a negative control. The proteins were separated by sodium dodecyl sulfate polyacrylamide gel electrophoresis (SDS-PAGE) under reducing or non-reducing conditions and transferred onto a nitrocellulose membrane using a Trans-Blot SD Semi-Dry Transfer Cell device (Bio-Rad). The nitrocellulose membranes were blocked for 1 h using 5% non-fat dry milk diluted in PBS and subsequently incubated with the hybridoma supernatants (diluted 1:20 in PBS) overnight at 4 °C followed by a horseradish peroxidase-conjugated anti-mouse antibody (Dako, diluted 1:200 in PBS) for 1 h at room temperature. Proteins were visualized using the Super Signal West Pico Chemiluminescent substrat (Thermo Scientific).

### PepScan analysis of anti-N monoclonal antibodies

A library of biotinylated overlapping peptides covering the entire N-protein sequence was synthesized (New England Peptide, USA). Biotinylated peptides were 16 amino acids (aa) long with an overlap of 13 aa. Synthesis was successful for all requested peptides except for peptides number B10 and D08 which were excluded from the analysis because they did not pass quality control (please see Additional file 1 for sequence information).

Biotinylated peptides were coated on 96-well streptavidin plates (IDEXX International, USA) and the N-specific mAbs were subsequently tested for binding activity on each peptide. In addition, binding activity of full-length recombinant N-protein (rN) was assessed as a control on the same plate. The rN-protein was cloned in pET28a and expressed in E. coli BL21 cells (Novagen, UK). Water soluble His-tagged protein was purified by affinity chromatography on Ni^2+^-columns [21] and biotinylated subsequently (EZ-Link® Sulfo-NHS-LC-Biotinylation Kit, Thermo Scientific). The indirect ELISA was performed as follows: Streptavidin plates were washed three times with PBS containing 0.1% Tween-20. Peptides (5.0 to 7.5 mg/mL in DMSO) were diluted 1:400 in PBS pH 7.2 and coated using 100 μL/well during 1 h at room temperature. Plates were then washed three times using washing buffer (PBS containing 0.1% Tween-20). Subsequently, 100 μL of the diluted hybridoma supernatants were added to each well and incubated for 1 h at room temperature. After three additional washing steps, 100 μL/well of goat anti-mouse peroxidase-conjugated antibody (Bio-Rad) were added and incubated for 1 h at room temperature. Dilutions were both prepared in PBS containing 0.1% Tween-20 and 10% goat serum. Finally, the plates were washed three times with washing buffer and subsequently 3,3′,5,5’-tetramethylbenzidine (TMB substrate) was added to each well. The color development was stopped after 10 min and optical densities (OD) were measured at 450 nm using a spectrophotometer. When two contiguous peptides were recognized, it was assumed that the binding area was the common amino acid sequence of the two contiguous peptides.

The identified epitopes were highlighted in the three-dimensional structure of SBV-N [22] using the PyMOL software [23].

## Results

### MAb production

A panel of 47 hybridomas, generated by fusion processes from two immunized mice, was found to produce mAbs specifically reactive with homologous SBV in IIF and indirect ELISA tests.

In a preliminary characterization aimed at selection of mAbs for further studies, the 47 MAbs showed two different immunofluorescence patterns (described in the following paragraph), the majority reacted in western blot with either a band of approximately 25 kDa or a band of 110 kDa. The former did not show neutralizing activity, while most of the latter did neutralize SBV infectivity, suggesting a strong involvement of the target protein in virus neutralization mechanisms.

After preliminary characterization and diversification, a subset of 16 hybridomas was submitted to cloning procedures and the produced mAbs were used for the following more detailed investigations.

### Evaluation of the 16 mAbs in VNT, IIF test and Western blot analysis

In the IIF tests, six mAbs reacted with transiently expressed SBV-N (pCITE_N transfected cells) and ten with SBV-M (pCITE_M transfected cells) (Table 1).

**Table 1:** **Characterization of anti-SBV mAbs by immunofluorescence test**

**MAbs**	**indirect immunofluorescence test (IIFT)**															**IIFT pCITE clones**		
	**Schmallenberg virus**						**DOUV**	**SIMV**	**SATV**	**SHAV**	**SABOV**	**AKAV**	**AINOV**	**PEAV**	**OROV**	**Schmallenberg virus**		
	**BH80/11-4**	**BH619/12-3**	**BH652/12**	**D512/12**	**D495/12-1**	**D495/12-2**										**S**	**M**	**Gc**
4E12	+	+	+	+	+	+	+	+	+	+	+	+	-	-	-	+		
1H4	+	+	+	+	+	+	+	+	+	+	+	+	+	+	+	+		
3H3	+	+	+	+	+	+	+	+	+	+	+	+	-	-	-	+	-	
3E3	+	+	+	+	+	+	+	+	+	+	+	+	-	-	-	+		
3H9	+	+	+	+	+	+	+	-	+	+	-	-	-	-	-	+	-	
3A11	+	+	+	+	+	+	+	-	+	+	-	-	-	-	-	+		
2G10	+	+	+	+	+	+	-	-	-	-	-	-	-	-	-	-	+	+
4D9	+	+	+	+	+	+	-	-	-	-	-	-	-	-	-	-	+	+
2H11	+	+	+	+	+	+	+	-	+	-	-	-	-	-	-	-	+	+
4E5	+	+	+	+	+	+	-	-	-	-	-	-	-	-	-	-	+	+
5 F8	+	+	+	+	-	-	-	-	-	-	-	-	-	-	-		+	+
1C11	+	+	+	+	+	+	+	-	+	-	-	-	-	-	-		+	+
1 F4	+	+	+	+	+	+	-	-	+	-	-	-	-	-	-		+	+
4B6	+	+	+	+	+	+	-	-	-	-	-	-	-	-	-	-	+	+
1C1	+	+	+	+	+	+	+	-	+	-	-	-	-	-	-	-	+	+
3A5	+	+	+	+	+	+	+	-	+	-	-	-	-	-	-	-	+	+

**Figure 1:**
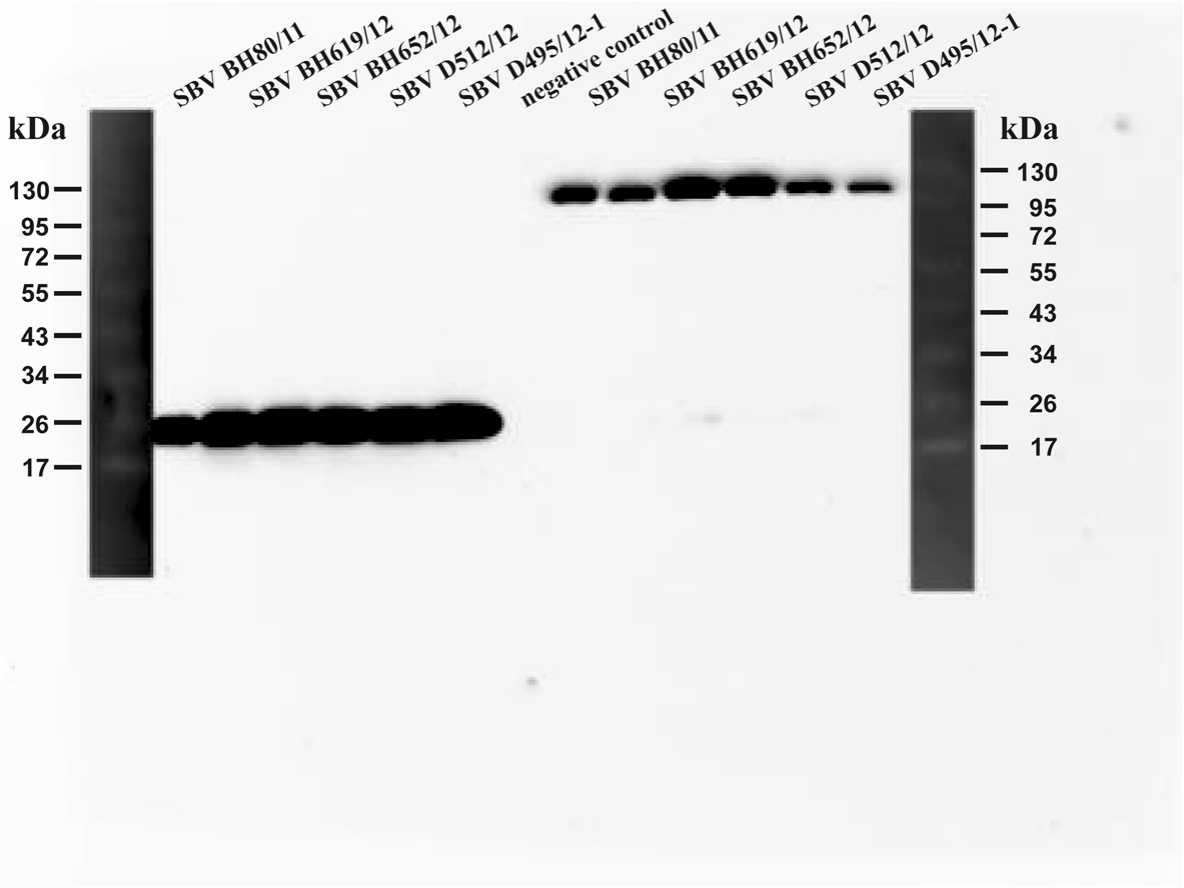
**Western blot analysis of mAbs 4E12 and 1F4.** The anti-N mAb 4E12 is shown in lanes 1 to 7, and mAb 1F4 (anti-Gc) in lanes 7 to 13.

When analyzed in WB (non-reducing conditions), using the homologous strain BH80/11, four out of the six SBV-N-reactive mAbs bound a protein of the approximate molecular weight of the N-protein (25 kDa, Figure 1). The remaining two mAbs (3H9 and 3A11) did not recognize any denatured SBV antigen in cell lysates (Table 2). None of the six mAbs was reactive under reducing conditions (data not shown). The 10 mAbs which reacted with pCITE_M transfected cells detected a Gc-specific band of about 110 kDa in WB (Table 2 and Figure 1).

**Table 2:** **Characterization of anti-SBV mAbs by ELISA, neutralization test, and western blot**

**MAbs**	**ELISA**	**VNT**	**Western blot (kDa)**														
			**Schmallenberg virus**						**DOUV**	**SIMV**	**SATV**	**SHAV**	**SABOV**	**AKAV**	**AINOV**	**OROV**	
			**BH80/11-4**	**BH619/12-3**	**BH652/12**	**D512/12**	**D495/12-1**	**D495/12-2**									**Specifity**
4E12	+	<1/2	~25	~25	~25	~25	~25	~25	~25	~25	~25	~25	~25	~25	-	-	**nucleocapsid protein**
1H4	+	<1/2	~25	~25	~25	~25	~25	~25	~25	~25	~25	~25	~25	~25	-	~25	**nucleocapsid protein**
3H3	+	<1/2	~25	~25	~25	~25	~25	~25	~25	~25	~25	~25	~25	~25	-	-	**nucleocapsid protein**
3E3	+	<1/2	~25	~25	~25	~25	~25	~25	~25	~25	~25	~25	-	-	-	-	**nucleocapsid protein**
3H9	+	<1/2	-	-	-	-	-	-		-	-	-	-				**nucleocapsid protein**
3A11	+	<1/2	-	-	-	-	-	-		-	-	-	-				**nucleocapsid protein**
2G10	+	<1/2	~110	~110	~110	~110	~110	~110									**glycoprotein Gc**
4D9	+	pos	~110	~110	~110	~110	~110	~110									**glycoprotein Gc**
2H11	+	<1/2	~110	~110	~110	~110	~110	~110	~110	-	-	-	-				**glycoprotein Gc**
4E5	+	pos	~110	~110	~110	~110	~110	~110									**glycoprotein Gc**
5 F8	+	1/8	~110	-	-	-	-	-									**glycoprotein Gc**
1C11	+	1/16	~110	~110	~110	~110	~110	~110	-	-	~110	-	-				**glycoprotein Gc**
1 F4	+	1/4	~110	~110	~110	~110	~110	~110	-	-	-	-	-				**glycoprotein Gc**
4B6	+	1/8	~110	~110	~110	~110	~110	~110									**glycoprotein Gc**
1C1	+	1/16	~110	~110	~110	~110	~110	~110		-	~110	-	-				**glycoprotein Gc**
3A5	+	<1/2	~110	~110	~110	~110	~110	~110		-	~110	-	-				**glycoprotein Gc**

**Figure 2:**
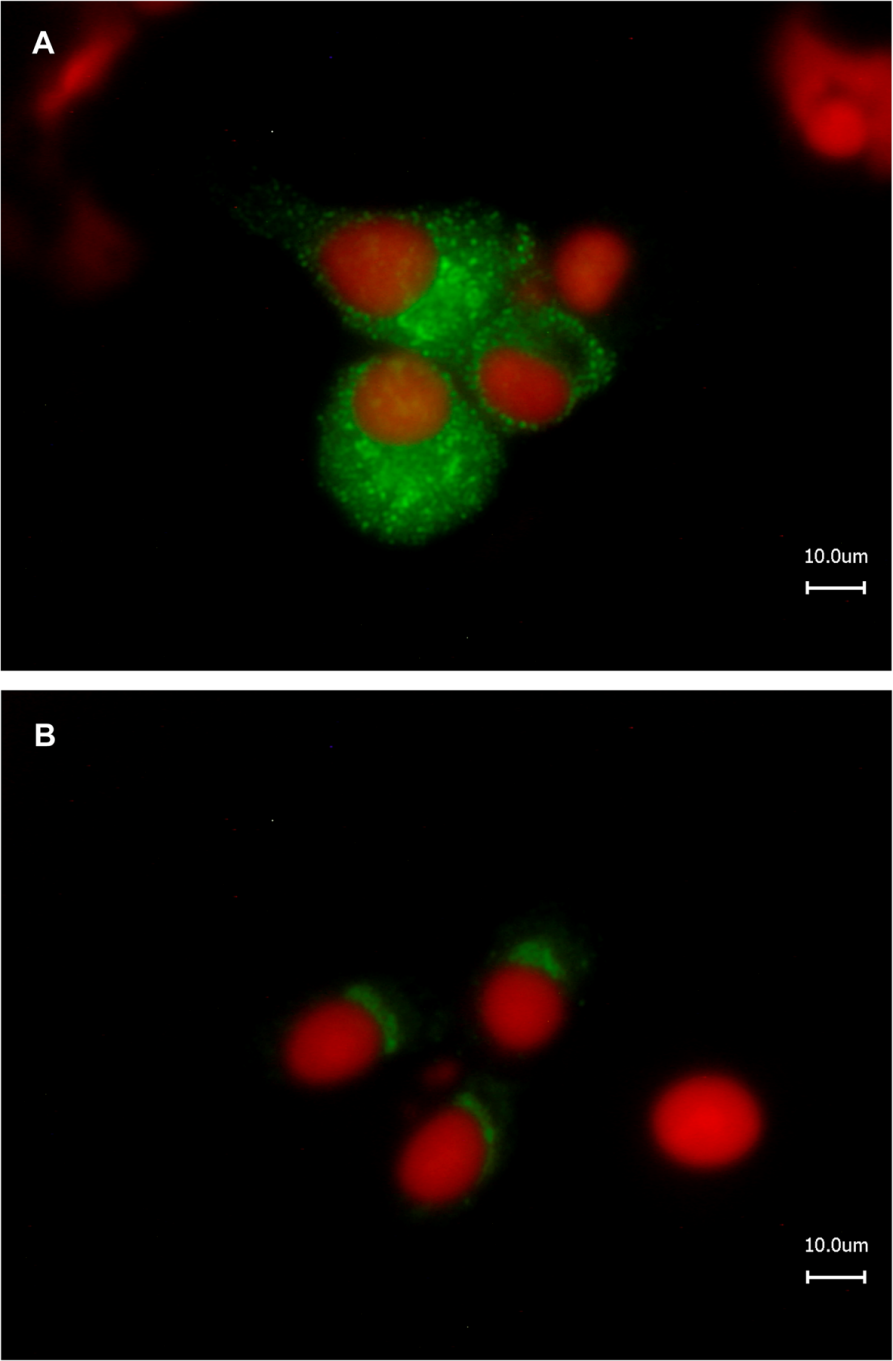
**Indirect immunofluorescence test.** MAbs 4E12 (anti-N, **A**) and 1F4 (anti-Gc, **B**) were analyzed in an indirect immunofluorescence test using BHK-21 cells infected with SBV strain BH80/11 as antigen matrix.

All mAbs showed a strong reactivity in BHK21-cells infected with the homologous SBV strain BH80/11. However, the individual mAbs showed different fluorescence staining patterns. By using the SBV-N-reactive mAbs a cytoplasmatic fluorescence and granular inclusions could be observed (see Figure 2A for an example). In contrast, a more diffuse, homogenous immunofluorescence around the nuclei of the infected cells was produced by SBV-M-reactive mAbs (Figure 2B).

Eventually, the target-specificity of the mAbs was determined by combining the results of WB analysis and IIF tests described above: 6 out of the 16 characterized mAbs were specific for the N-protein, and 10 specifically reacted with Gc. Investigation of the virus neutralizing capability by VNT showed that none of the six anti-N mAbs was able to neutralize virus infectivity, in contrast to seven out of 10 anti-Gc mAbs, which demonstrated neutralizing activity (Table 2).

Apart from the homologous strain BH80/11, five additional SBV isolates were used to investigate the reactivity of the mAbs. The results of both, WB analysis and IIF tests were in agreement for all isolates except for mAb 5 F8 (Tables 1 and 2). In IIF, this antibody detected 4 out of 6 SBV isolates, whereas in WB, it reacted only with the isolate BH80/11-4, which was used for the mAb production.

**Figure 3:**
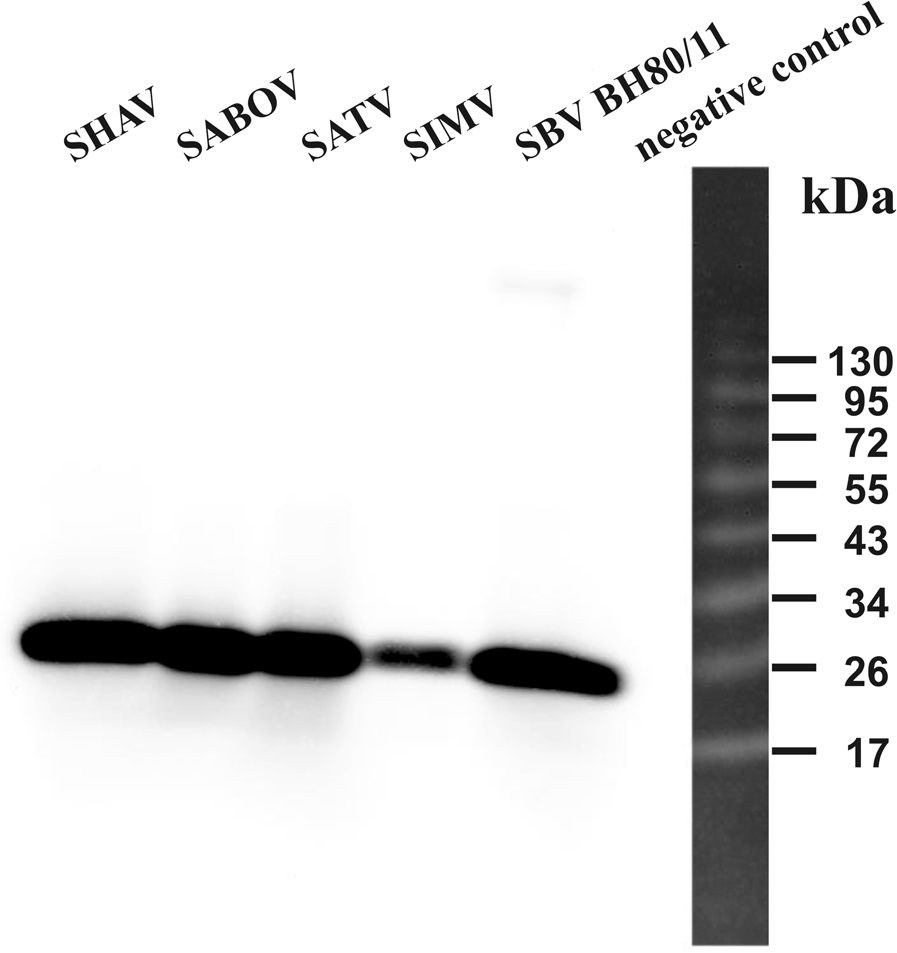
**Western blot analysis of mAbs 4E12 with Simbu serogroup viruses.** Mock-infected BHK-21 cells were used as negative control.

The reactivity of the mAbs with nine related Simbu viruses was subsequently also investigated. The N-specific mAbs were able to detect DOUV, SATV, and SHAV infected cells (see Figure 3 for an example) and four out of six mAbs reacted also with SIMV, SABOV, and AKAV infected cells in IIF tests. The mAb 1H4 even showed a pan-Simbu reactivity as it detected all of the tested Simbu serogroup viruses. Of the Gc-specific mAbs, one detected SATV and four recognized SATV as well as DOUV. However, the remaining 5 mAbs did not recognize any of the tested Simbu viruses (Table 1). In WB, all the SBV N-specific mAbs except 3H9 and 3A11 reacted with DOUV, SIMV, SATV, and SHAV, three of them also detected SABOV and AKAV, and mAb 1H4 showed reactivity with all tested Simbu viruses except AINOV. Among the Gc-specific mAbs, one showed positive signals with DOUV and another three were able to detect SATV (Table 2).

### Epitope mapping and location on the three-dimensional structure of anti-N monoclonal antibodies

The anti-N mAbs showed the broadest reactivity spectrum against all the tested bunyaviruses, hence determining the relevant epitopes could be helpful to explain the observed patterns. For this purpose, a PepScan approach was applied, in which overlapping peptides that covered the complete sequence of the N‐protein were synthesized and tested with the respective mAbs in an ELISA.

**Figure 4:**
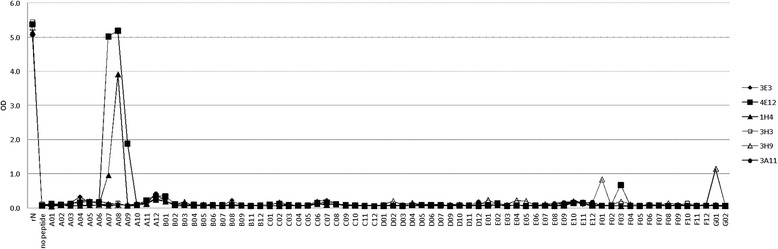
**PepScan analysis of the anti-N antibodies.** Binding of mAbs 3E3 clone 10 (filled diamond), 4E12 clone 9 (filled square), 1H4 clone 7 (filled triangle), 3H3 clone 2 (open square) 3H9 clone 4 (open triangle), and 3A11 clone 2 (filled circle) to full length N-protein (rNP) and to N-protein (Np) peptides.

**Figure 5:**
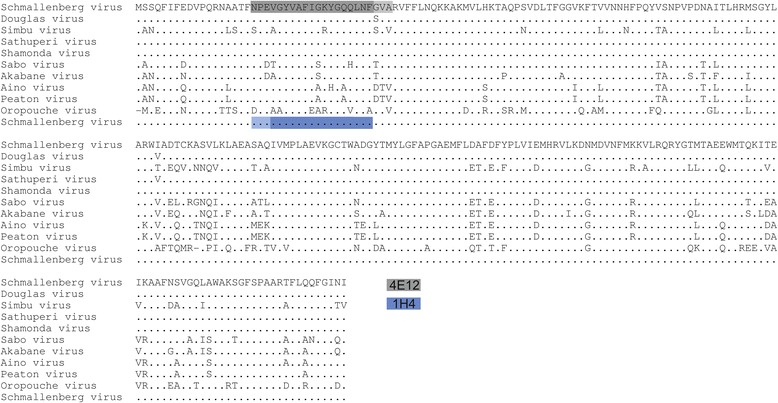
**Sequence alignment of N-protein of Simbu serogroup viruses.** Amino acids that match those of SBV are indicated by dots. The linear epitopes identified by using pepscanning are highlighted, a strong ELISA reaction is represented in dark grey (mAb 4E12) or dark blue (mAb 1H4), whereas a weaker reaction is indicated in light grey (4E12) or light blue (1H4).

The mAbs 4E12 and 1H4 showed a strong and reproducible binding activity to some contiguous peptides allowing the localization of the target epitope (Figures 4 and 5). MAb 4E12 bound strongly to the peptides A07, A08 (OD > 5.0) and to a lower extent to A09 (OD 1.9). MAb 1H4 bound strongly to the peptide A08 (OD 3.9) and on a clearly lower level to A07 (OD 1.0). Since peptides A07 (aa 19–34), A08 (aa 22–37) and A09 (aa 25–40) are contiguous peptides within the N-protein of SBV strain BH80/11 (Figure 5) it could be assumed that this aa region represents the binding area of the mAbs 4E12 and 1H4 or is at least a part of it. However, for the remaining four N-specific mAbs, no binding activity could be detected in the peptide ELISA although all mAbs strongly bound to the full length N-protein (Figure 4). Two of these four mAbs showed also no reactivity in WB-analyses, indicating a conformation-dependent nature of their target epitopes.

The reactions of 3E3 and 4E12 with peptide F03 and of 3H9 with peptides F01 and G01 could not be confirmed and are possibly due to conjugate artifacts.

**Figure 6:**
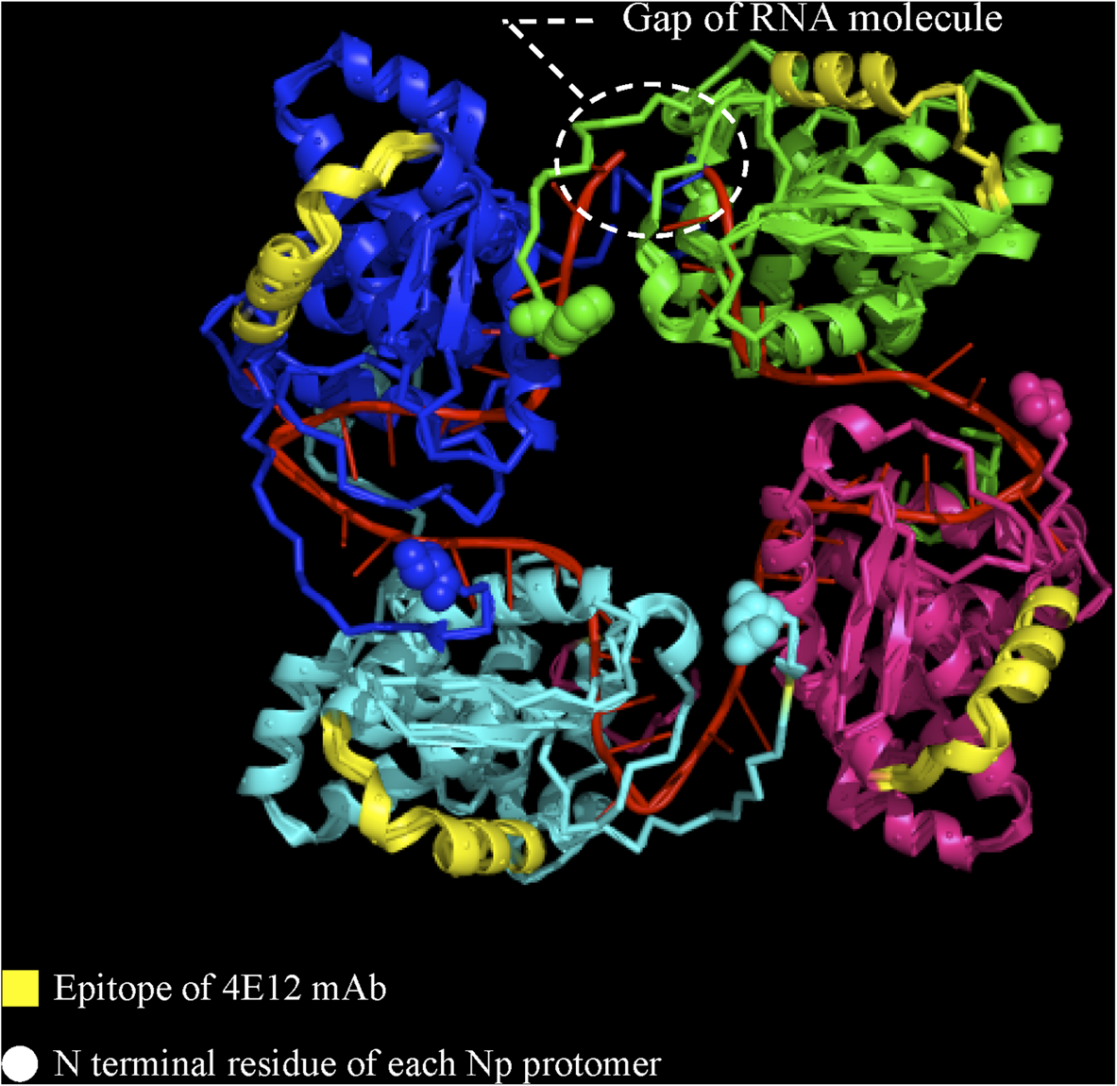
**Location of the antigenic site of mAb 4E12 in the three-dimensional structure of SBV-N.** The structure of SBV-N was obtained from [22]. This figure was prepared using the PyMOL program [23].

The epitope formed by peptides A07, A08, and A09 corresponds to amino acid positions 19 to 40 of SBV strain BH80/11 and could be localized at the external face of the N-protein tetramer in the crystal structure of SBV‐N [22] (Figure 6).

## Discussion

The Simbu serogroup of the genus *Orthobunyavirus* contains viruses of different medical and veterinary relevance and members of this group have been isolated worldwide [24]. OROV, the only virus in the serogroup that can infect humans, was responsible for outbreaks of acute febrile illness in South America [25,26]. AKAV, a serious threat to the livestock industry, caused epizootics of congenital defects in Asia, Australia, or in the Middle East [27-30]. An AKAV-infection during a critical period of gestation can lead to the birth of severely malformed offspring, premature birth or stillbirth. Identical symptoms are induced by an infection of pregnant ruminants with other members of the Simbu serogroup as e.g. AINOV or SBV [30-34]. SBV, a novel orthobunyavirus and the first European member of this serogroup, was discovered for the first time in late 2011. Since then, the virus caused a large epidemic in domestic and wild ruminants all over Europe [35-37]. To enable early detection of an SBV-spread into previously unaffected regions and to monitor emergence of other Simbu serogroup viruses, sensitive and specific diagnostic test systems are required.

The N-protein represents the most abundant protein in the virion as well as in virus-infected cells and is currently widely used for serological detection of SBV infections [16,17]. In addition, the N-protein encoding gene provides a reliable target of SBV molecular diagnostics tests. Furthermore, due to its high immunogenicity, N-specific antibodies are detectable early after infection as well as in convalescent animals providing a reliable basis for serological diagnosis [38,39]. However, due to the high sequence conservation of the N-gene, antibody cross-reactivity occurs among members of the same serogroup [17,40,41]. Accordingly, the N-specific mAbs produced in the present study recognized several viruses of the Simbu serogroup (Tables 1 and 2). Nevertheless, only the mAb 1H4 allowed detection of all the tested virus strains in the immunofluorescence test. This antibody therefore represents a promising candidate for the development of serological screening tests which cover the entire Simbu serogroup. The mAb could e.g. be applied in a blocking or competing ELISA format.

Using PepScan analysis, the epitope of mAb 1H4 and 4E12 could be localized to amino acid positions 19 to 40 of the SBV BH80/11-N-protein. These results are not in agreement with a previously with another mAb identified antigenic epitope located between aa 51 to 76 [41]. However, the respective experiments have been performed using a bacterially expressed full-length recombinant SBV-N-protein, whereas a peptide library was applied in the present study, which might explain the diverging results. Using 3D modelling, the epitope recognized by mAbs 1H4 and 4E12 could be located to the external surface of the tetramer formed by the N‐protein in complex with the RNA molecule, rendering this domain accessible to the humoral immune system. However, even though these two mAbs bound to the same epitope in the PepScan analysis, they showed differences in their reactivity with Simbu serogroup viruses in IIF and WB. It can therefore be assumed that mAbs 1H4 and 4E12 bind to different key amino acid residues within overlapping linear epitopes.

For several of the mAbs used in this study a specific epitope could not be identified, indicating that the respective antibodies bind to discontinuous epitopes [42]. In such cases, the antibodies are not directed against a linear stretch of aa, but recognize a set of amino acid residues in the antigen that are brought together only by the folding of the molecule [42]. Since with the applied PepScan technology only linear epitopes can be mapped, it is not suitable to investigate conformation-dependent epitopes. This might explain the failure to define epitopes for each of the antibodies.

Virus neutralization activity was observed for none of the generated anti-N mAbs, which is in agreement with previous studies. Although N represents the immunodominant protein in several members of the *Bunyaviridae* family, it has been shown before, that anti-N antibodies usually do not have neutralizing activity. In contrast, almost all neutralizing antibodies are directed against the glycoproteins [9,43-45]. This is in accordance with the results of the present study: 7 out of 10 mAbs specific for M-segment encoded proteins were positive in the VNT. Furthermore, all the isolated glycoprotein-specific antibodies were directed against Gc, suggesting that the virus particle‐associated Gc is more immunogenic than Gn. It has already been shown before in immunization experiments, e.g. using the bunyavirus LaCrosse virus, that the Gc-protein can be the main target of the humoral immune response [9,46].

In contrast to the conserved S-segment, the M-segment is variable between different SBV strains and between different virus species of the Simbu serogroup. A region of especially high sequence variability was recently described within the gene encoding Gc [13,14]. Consequently, five out of the 10 Gc-specific mAbs exclusively detected SBV isolates in the present study. The remaining five mAbs additionally recognized DOUV and SATV, which are the most closely related viruses to SBV based on M-segment sequences [2]. However, cross-reactivity against other Simbu serogroup viruses was not observed.

Interestingly, two of the mAbs which detected SBV as well as DOUV and SATV (mAbs 1C1 and 1C11) are possibly directed against the same epitope within the Gc-glycoprotein. This was recently suggested based on their identical reactivity profile with an escape-mutant SBV (resistant to neutralization) which was generated by mAb 1C11 (Brocchi and Cordioli, personal communication). In addition, the mAb 1C11 (or alternatively 1C1) has already been successfully employed as competitor-conjugated mAb in a competitive ELISA which allows antibody detection in different species [47].

The mAb 2H11 did not bind as strong as the other Gc-mAbs to Gc-protein expressed in transfected cells, even though it recognized a product of the approximate molecular size of the mature Gc in western blot and reacted with virus-infected cells in the IIF test. Maybe, this antibody recognizes the SBV-Gc better in a conformation which relies on the presence of the Gn-protein indicating that the mAb either reacts with the Gn‐Gc complex and/or with the Gc alone once it is chaperoned by Gn. This hypothesis is supported by previous studies in Bunyamwera virus which demonstrated the importance of Gn in the transport of Gc to the Golgi and in Gc-Gn-heterodimerization [48,49].

Another intriguing finding of the present study was the highly specific binding pattern of the Gc-specific mAb 5F8 which reacted almost exclusively with the homologous SBV strain BH80/11. Similar observations have previously been described for AKAV- and AINOV-specific mAbs [50,51]. However, the respective virus strains were isolated during a period of more than 30 years (AKAV from 1959 to 1990, and AINOV between 1964 and 1995) on two different continents, which explains the observed antigenic differences. In contrast, the SBV strains used in the present study were collected only during 2011 or 2012 and in a spatially limited area. Nevertheless, different mAb-reactivity patterns were obtained for these isolates. Thus, the mAbs investigated in this study provide a valuable tool to rapidly determine the phenotype of SBV isolates and to characterize newly emerging virus strains. Furthermore, they represent attractive options for the development of novel immunodiagnostic assays which might allow differentiation of SBV and other Simbu serogroup viruses.

## Additional file

Additional file 1:
**Sequences of peptides synthesized for SBV N-protein.** A library of biotinylated overlapping peptides covering the entire N-protein sequence was synthesized, the peptides were 16 amino acids (aa) long with an overlap of 13 aa.

## References

[1] Hoffmann B, Scheuch M, Höper D, Jungblut R, Holsteg M, Schirrmeier H, Eschbaumer M, Goller KV, Wernike K, Fischer M, Breithaupt A, Mettenleiter TC, Beer M (2012). Novel orthobunyavirus in cattle, Europe, 2011. Emerg Infect Dis.

[2] Goller KV, Höper D, Schirrmeier H, Mettenleiter TC, Beer M (2012). Schmallenberg virus as possible ancestor of Shamonda virus. Emerg Infect Dis.

[3] Yanase T, Kato T, Aizawa M, Shuto Y, Shirafuji H, Yamakawa M, Tsuda T (2012). Genetic reassortment between Sathuperi and Shamonda viruses of the genus Orthobunyavirus in nature: implications for their genetic relationship to Schmallenberg virus. Arch Virol.

[4] Guu TS, Zheng W, Tao YJ (2012). Bunyavirus: structure and replication. Adv Exp Med Biol.

[5] Walter CT, Barr JN (2011). Recent advances in the molecular and cellular biology of bunyaviruses. J Gen Virol.

[6] Chowdhary R, Street C, Travassos da Rosa A, Nunes MR, Tee KK, Hutchison SK, Vasconcelos PF, Tesh RB, Lipkin WI, Briese T (2012). Genetic characterization of the Wyeomyia group of orthobunyaviruses and their phylogenetic relationships. J Gen Virol.

[7] Dong H, Li P, Elliott RM, Dong C (2013). Structure of Schmallenberg orthobunyavirus nucleoprotein suggests a novel mechanism of genome encapsidation. J Virol.

[8] Elliott RM, Blakqori G, Plyusnin A, Elliott RM (2011). Molecular Biology of Orthobunyaviruses. Bunyaviridae Molecular and Cellular Biology.

[9] Gonzalez-Scarano F, Shope RE, Calisher CE, Nathanson N (1982). Characterization of monoclonal antibodies against the G1 and N proteins of LaCrosse and Tahyna, two California serogroup bunyaviruses. Virology.

[10] Meroc E, Poskin A, Van Loo H, Quinet C, Van Driessche E, Delooz L, Behaeghel I, Riocreux F, Hooyberghs J, De Regge N, Caij AB, Van den Berg T, Van der Stede Y (2013). Large-scale cross-sectional serological survey of Schmallenberg virus in Belgian cattle at the end of the first vector season. Transbound Emerg Dis.

[11] Wernike K, Silaghi C, Nieder M, Pfeffer M, Beer M (2014). Dynamics of Schmallenberg virus infection within a cattle herd in Germany, 2011. Epidemiol Infect.

[12] Kobayashi T, Yanase T, Yamakawa M, Kato T, Yoshida K, Tsuda T (2007). Genetic diversity and reassortments among Akabane virus field isolates. Virus Res.

[13] Fischer M, Hoffmann B, Goller KV, Höper D, Wernike K, Beer M (2013). A mutation ‘hot spot’ in the Schmallenberg virus M segment. J Gen Virol.

[14] Coupeau D, Claine F, Wiggers L, Kirschvink N, Muylkens B (2013). In vivo and in vitro identification of a hypervariable region in Schmallenberg virus. J Gen Virol.

[15] Hulst M, Kortekaas J, Hakze-van der Honing R, Vastenhouw S, Cornellissen J, van Maanen K, Bossers A, Harders F, Stockhofe N, van der Poel W (2013). Genetic characterization of an atypical Schmallenberg virus isolated from the brain of a malformed lamb. Virus Genes.

[16] Bilk S, Schulze C, Fischer M, Beer M, Hlinak A, Hoffmann B (2012). Organ distribution of Schmallenberg virus RNA in malformed newborns. Vet Microbiol.

[17] Breard E, Lara E, Comtet L, Viarouge C, Doceul V, Desprat A, Vitour D, Pozzi N, Cay AB, De Regge N, Pourquier P, Schirrmeier H, Hoffmann B, Beer M, Sailleau C, Zientara S (2013). Validation of a commercially available indirect ELISA using a nucleocapside recombinant protein for detection of Schmallenberg virus antibodies. PLoS One.

[18] Galfrè G, Milstein C (1981). Preparation of monoclonal antibodies: strategies and procedures. Methods Enzymol.

[19] Buchholz UJ, Finke S, Conzelmann KK (1999). Generation of bovine respiratory syncytial virus (BRSV) from cDNA: BRSV NS2 is not essential for virus replication in tissue culture, and the human RSV leader region acts as a functional BRSV genome promoter. J Virol.

[20] Geiser M, Cebe R, Drewello D, Schmitz R (2001). Integration of PCR fragments at any specific site within cloning vectors without the use of restriction enzymes and DNA ligase. Biotechniques.

[21] Block H, Maertens B, Spriestersbach A, Brinker N, Kubicek J, Fabis R, Labahn J, Schafer F (2009). Immobilized-metal affinity chromatography (IMAC): a review. Methods Enzymol.

[22] Dong H, Li P, Bottcher B, Elliott RM, Dong C (2013). Crystal structure of Schmallenberg orthobunyavirus nucleoprotein-RNA complex reveals a novel RNA sequestration mechanism. RNA.

[23] DeLano WL (2002). The PyMOL user’s Manual.

[24] Saeed MF, Li L, Wang H, Weaver SC, Barrett AD (2001). Phylogeny of the Simbu serogroup of the genus Bunyavirus. J Gen Virol.

[25] Vasconcelos HB, Azevedo RS, Casseb SM, Nunes-Neto JP, Chiang JO, Cantuaria PC, Segura MN, Martins LC, Monteiro HA, Rodrigues SG, Nunes MR, Vasconcelos PF (2009). Oropouche fever epidemic in Northern Brazil: epidemiology and molecular characterization of isolates. J Clin Virol.

[26] Bastos Mde S, Figueiredo LT, Naveca FG, Monte RL, Lessa N, Pinto de Figueiredo RM, Gimaque JB, Pivoto Joao G, Ramasawmy R, Mourao MP (2012). Identification of Oropouche Orthobunyavirus in the cerebrospinal fluid of three patients in the Amazonas, Brazil. Am J Trop Med Hyg.

[27] Brenner J, Tsuda T, Yadin H, Chai D, Stram Y, Kato T (2004). Serological and clinical evidence of a teratogenic Simbu serogroup virus infection of cattle in Israel, 2001–2003. Vet Ital.

[28] Inaba Y, Kurogi H, Omori T (1975). Letter: Akabane disease: epizootic abortion, premature birth, stillbirth and congenital arthrogryposis-hydranencephaly in cattle, sheep and goats caused by Akabane virus. Aust Vet J.

[29] Kurogi H, Inaba Y, Goto Y, Miura Y, Takahashi H (1975). Serologic evidence for etiologic role of Akabane virus in epizootic abortion-arthrogryposis-hydranencephaly in cattle in Japan, 1972–1974. Arch Virol.

[30] Kirkland PD, Barry RD, Harper PA, Zelski RZ (1988). The development of Akabane virus-induced congenital abnormalities in cattle. Vet Rec.

[31] Noda Y, Uchinuno Y, Shirakawa H, Nagasue S, Nagano N, Ohe R, Narita M (1998). Aino virus antigen in brain lesions of a naturally aborted bovine fetus. Vet Pathol.

[32] Uchinuno Y, Noda Y, Ishibashi K, Nagasue S, Shirakawa H, Nagano M, Ohe R (1998). Isolation of Aino virus from an aborted bovine fetus. J Vet Med Sci.

[33] Konno S, Moriwaki M, Nakagawa M (1982). Akabane disease in cattle: congenital abnormalities caused by viral infection. Spontaneous disease. Vet Pathol.

[34] Conraths FJ, Peters M, Beer M (2013). Schmallenberg virus, a novel orthobunyavirus infection in ruminants in Europe: potential global impact and preventive measures. N Z Vet J.

[35] EFSA. “Schmallenberg” virus: analysis of the epidemiological data (November 2012). EFSA Supporting Publications 2012 EN-360 http://www.efsa.europa.eu/en/supporting/doc/360e.pdf. Accessed 15/07/2013.

[36] EFSA. “Schmallenberg” virus: analysis of the epidemiological data (May 2013). EFSA Supporting Publications 2013 EN-3429 http://www.efsa.europa.eu/de/supporting/doc/429e.pdf. Accessed 15/07/2013.

[37] Wernike K, Conraths F, Zanella G, Granzow H, Gache K, Schirrmeier H, Valas S, Staubach C, Marianneau P, Kraatz F, Höreth-Böntgen D, Reimann I, Zientara S, Beer M (2014). Schmallenberg virus-two years of experiences. Prev Vet Med.

[38] Fafetine JM, Tijhaar E, Paweska JT, Neves LC, Hendriks J, Swanepoel R, Coetzer JA, Egberink HF, Rutten VP (2007). Cloning and expression of Rift Valley fever virus nucleocapsid (N) protein and evaluation of a N-protein based indirect ELISA for the detection of specific IgG and IgM antibodies in domestic ruminants. Vet Microbiol.

[39] Jansen van Vuren P, Potgieter AC, Paweska JT, van Dijk AA (2007). Preparation and evaluation of a recombinant Rift Valley fever virus N protein for the detection of IgG and IgM antibodies in humans and animals by indirect ELISA. J Virol Methods.

[40] Fischer M, Schirrmeier H, Wernike K, Wegelt A, Beer M, Hoffmann B (2013). Development of a pan-Simbu real-time reverse transcriptase PCR for the detection of Simbu serogroup viruses and comparison with SBV diagnostic PCR systems. Virol J.

[41] Zhang Y, Wu S, Wang J, Wernike K, Lv J, Feng C, Zhang J, Wang C, Deng J, Yuan X, Lin X (2013). Expression and purification of the nucleocapsid protein of Schmallenberg virus, and preparation and characterization of a monoclonal antibody against this protein. Protein Expr Purif.

[42] Van Regenmortel MH (1989). Structural and functional approaches to the study of protein antigenicity. Immunol Today.

[43] Yu L, Zhang L, Sun L, Lu J, Wu W, Li C, Zhang Q, Zhang F, Jin C, Wang X, Bi Z, Li D, Liang M (2012). Critical epitopes in the nucleocapsid protein of SFTS virus recognized by a panel of SFTS patients derived human monoclonal antibodies. PLoS One.

[44] Magurano F, Nicoletti L (1999). Humoral response in Toscana virus acute neurologic disease investigated by viral-protein-specific immunoassays. Clin Diagn Lab Immunol.

[45] Boshra H, Lorenzo G, Busquets N, Brun A (2011). Rift valley fever: recent insights into pathogenesis and prevention. J Virol.

[46] Pekosz A, Griot C, Stillmock K, Nathanson N, Gonzalez-Scarano F (1995). Protection from La Crosse virus encephalitis with recombinant glycoproteins: role of neutralizing anti-G1 antibodies. J Virol.

[47] Cordioli P, Lelli D, Moreno A, Pezzoni G, Gamba D, Canelli E, Brocchi E (2013). Competitive mAb-Based ELISA for the Detection of Antibodies Against Schmallenberg Virus.

[48] Lappin DF, Nakitare GW, Palfreyman JW, Elliott RM (1994). Localization of Bunyamwera bunyavirus G1 glycoprotein to the Golgi requires association with G2 but not with NSm. J Gen Virol.

[49] Shi X, Lappin DF, Elliott RM (2004). Mapping the Golgi targeting and retention signal of Bunyamwera virus glycoproteins. J Virol.

[50] Yoshida K, Ohashi S, Kubo T, Tsuda T (2000). Comparison of intertypic antigenicity of Aino virus isolates by dot immunobinding assay using neutralizing monoclonal antibodies. J Clin Microbiol.

[51] Akashi H, Inaba Y (1997). Antigenic diversity of Akabane virus detected by monoclonal antibodies. Virus Res.

