# Supplementary material for: A novel panel of monoclonal antibodies against Schmallenberg virus nucleoprotein and glycoprotein Gc allows specific orthobunyavirus detection and reveals antigenic differences
Source: Vet Res. 2015 Mar 11;46:27. doi: 10.1186/s13567-015-0165-4 (PMC4354985; doi:10.1186/s13567-015-0165-4)
Supplement: Additional file 1: — Sequences of peptides synthesized for SBV N-protein. A library of biotinylated overlapping peptides covering the entire N-protein sequence was synthesized, the peptides were 16 amino acids (aa) long with an overlap of 13 aa. [file 13567_2015_165_MOESM1_ESM.doc]

| **Short ID** | **Peptide Sequence** |  | **Short ID** | **Peptide Sequence** |
| --- | --- | --- | --- | --- |
| A01 | MSSQFIFEDVPQRNAA |  | D02 | VLKLAEASAQIVMPLA |
| A02 | QFIFEDVPQRNAATFN |  | D03 | LAEASAQIVMPLAEVK |
| A03 | FEDVPQRNAATFNPEV |  | D04 | ASAQIVMPLAEVKGCT |
| A04 | VPQRNAATFNPEVGYV |  | D05 | QIVMPLAEVKGCTWAD |
| A05 | RNAATFNPEVGYVAFI |  | D06 | MPLAEVKGCTWADGYT |
| A06 | ATFNPEVGYVAFIGKY |  | D07 | AEVKGCTWADGYTMYL |
| A07 | NPEVGYVAFIGKYGQQ |  | D08 | KGCTWADGYTMYLGFA* |
| A08 | VGYVAFIGKYGQQLNF |  | D09 | TWADGYTMYLGFAPGA |
| A09 | VAFIGKYGQQLNFGVA |  | D10 | DGYTMYLGFAPGAEMF |
| A10 | IGKYGQQLNFGVARVF |  | D11 | TMYLGFAPGAEMFLDA |
| A11 | YGQQLNFGVARVFFLN |  | D12 | LGFAPGAEMFLDAFDF |
| A12 | QLNFGVARVFFLNQKK |  | E01 | APGAEMFLDAFDFYPL |
| B01 | FGVARVFFLNQKKAKM |  | E02 | AEMFLDAFDFYPLVIE |
| B02 | ARVFFLNQKKAKMVLH |  | E03 | FLDAFDFYPLVIEMHR |
| B03 | FFLNQKKAKMVLHKTA |  | E04 | AFDFYPLVIEMHRVLK |
| B04 | NQKKAKMVLHKTAQPS |  | E05 | FYPLVIEMHRVLKDNM |
| B05 | KAKMVLHKTAQPSVDL |  | E06 | LVIEMHRVLKDNMDVN |
| B06 | MVLHKTAQPSVDLTFG |  | E07 | EMHRVLKDNMDVNFMK |
| B07 | HKTAQPSVDLTFGGVK |  | E08 | RVLKDNMDVNFMKKVL |
| B08 | AQPSVDLTFGGVKFTV |  | E09 | KDNMDVNFMKKVLRQR |
| B09 | SVDLTFGGVKFTVVNN |  | E10 | MDVNFMKKVLRQRYGT |
| B10 | LTFGGVKFTVVNNHFP* |  | E11 | NFMKKVLRQRYGTMTA |
| B11 | GGVKFTVVNNHFPQYV |  | E12 | KKVLRQRYGTMTAEEW |
| B12 | KFTVVNNHFPQYVSNP |  | F01 | LRQRYGTMTAEEWMTQ |
| C01 | VVNNHFPQYVSNPVPD |  | F02 | RYGTMTAEEWMTQKIT |
| C02 | NHFPQYVSNPVPDNAI |  | F03 | TMTAEEWMTQKITEIK |
| C03 | PQYVSNPVPDNAITLH |  | F04 | AEEWMTQKITEIKAAF |
| C04 | VSNPVPDNAITLHRMS |  | F05 | WMTQKITEIKAAFNSV |
| C05 | PVPDNAITLHRMSGYL |  | F06 | QKITEIKAAFNSVGQL |
| C06 | DNAITLHRMSGYLARW |  | F07 | TEIKAAFNSVGQLAWA |
| C07 | ITLHRMSGYLARWIAD |  | F08 | KAAFNSVGQLAWAKSG |
| C08 | HRMSGYLARWIADTCK |  | F09 | FNSVGQLAWAKSGFSP |
| C09 | SGYLARWIADTCKASV |  | F10 | VGQLAWAKSGFSPAAR |
| C10 | LARWIADTCKASVLKL |  | F11 | LAWAKSGFSPAARTFL |
| C11 | WIADTCKASVLKLAEA |  | F12 | AKSGFSPAARTFLQQF |
| C12 | DTCKASVLKLAEASAQ |  | G01 | GFSPAARTFLQQFGIN |
| D01 | KASVLKLAEASAQIVM |  | G02 | PAARTFLQQFGINI |
| *Synthesis of peptide failed | |  |  |  |
